# Editorial: Highly efficient selections and applicable utilizations of aptamers as molecular recognition elements

**DOI:** 10.3389/fchem.2026.1784694

**Published:** 2026-03-03

**Authors:** Xinhui Lou, Jiefang Sun, W. Andy Tao, Yiyang Dong

**Affiliations:** 1 Department of Chemistry, Capital Normal University, Beijing, China; 2 Beijing Key Laboratory of Diagnostic and Traceability Technologies for Food Poisoning, Beijing Center for Disease Prevention and Control, Beijing, China; 3 Department of Chemistry, Purdue University, West Lafayette, IN, United States; 4 College of Life Science and Technology, Beijing University of Chemical Technology, Beijing, China

**Keywords:** analytical chemistry, aptamer, aptasensor, biosensor, detection

Aptamers, invented in 1990s and often referred to as “chemical antibodies,” are single-stranded DNA or RNA oligonucleotides that bind to specific target molecules with high affinity and selectivity. These molecular recognition elements have emerged in recent years as powerful tools in various fields including detection, diagnostics, therapeutics, and biosensing. This Research Topic focuses on the latest advancements in aptamer selection methodologies and their practical applications, highlighting the growing importance of these versatile biomolecules.

The scope of the Research Topic​ brings together cutting-edge research on aptamer technology and representative applications, covering mainly the following three main areas.

- Novel selection techniques for high-efficiency aptamer identification
- Innovative biosensor development using aptamers as recognition elements
- Advanced applications for infectious disease control and food safety assurance

This Research Topic includes four featured articles as significant contributions that demonstrate the versatility and impact of aptamer technology.

“Application of DNA aptamers to block enterotoxigenic Escherichia coli toxicity in a Galleria mellonella larval model” - This study demonstrates the therapeutic potential of DNA aptamers in combating bacterial infections, using an innovative insect model system.

“Pyoverdine binding aptamers and label-free electrochemical detection of pseudomonads” - Presents a novel electrochemical biosensing platform for bacterial detection, showcasing the integration of aptamers with modern sensing technologies.

“Screening biotoxin aptamer and their application of optical aptasensor in food stuff: a review” - A comprehensive review that summarizes the technical points and difficulties of typical aptamer screening processes and the latest progresses in rapid optical detection of biotoxins for food safety applications.

“Recent research progress in tetrodotoxin detection and quantitative analysis methods” – A minireview that summarizes methodological updates of tetrodotoxin detection and quantitative analysis, covering frontier detection schemes based on biological and cellular sensors, immunoassays and immunosensors, aptamers, and liquid chromatography-mass spectrometry.

The articles presented in this Research Topic has already garnered significant attention, with over 13,000 article views and 12 citations, reflecting the broad readership and growing interest in aptamer technology. The contributions highlight how aptamers are transitioning from laboratory curiosities to practical tools with real-world applications as well.

As selection methodologies continue to improve and application platforms expand, we anticipate that aptamers will play an increasingly important role in molecular recognition. The integration of aptamers with advanced nanomaterials, microfluidics, and point-of-care devices promises to revolutionize present analytical chemistry.

